# Supplementary material for: Preliminary Evaluation of Bioactive Collagen–Polyphenol Surface Nanolayers on Titanium Implants: An X-ray Photoelectron Spectroscopy and Bone Implant Study
Source: J Funct Biomater. 2024 Jun 21;15(7):170. doi: 10.3390/jfb15070170 (PMC11278435; doi:10.3390/jfb15070170)
Supplement: Supplementary file 1 [file jfb-15-00170-s001.zip › jfb-2981092-supplementary.pdf]

Supplementary Materials

Preliminary Evaluation of Bioactive Collagen–Polyphenol Surface Nanolayers on Titanium Implants: An X-ray Photoelectron Spectroscopy and Bone Implant Study

Marco Morra [1,*], Giorgio Iviglia [1], Clara Cassinelli [1], Maria Sartori [2], Luca Cavazza [2], Lucia Martini [2], Milena Fini [3] and Gianluca Giavaresi [2]

[1] Nobil Bio Ricerche srl, V. Valcastellana 26, 14037 Portacomaro, Italy; giviglia@nobilbio.it (G.I.); ccassinelli@nobilbio.it (C.C.)
[2] Scienze e Tecnologie Chirurgiche, IRCCS Istituto Ortopedico Rizzoli, Via di Barbiano, 1/10, 40136 Bologna, Italy; maria.sartori@ior.it (M.S.); luca.cavazza@ior.it (L.C.); lucia.martini@ior.it (L.M.); gianluca.giavaresi@ior.it (G.G.)
[3] Direzione Scientifica, IRCCS Istituto Ortopedico Rizzoli, Via di Barbiano, 1/10, 40136 Bologna, Italy; milena.fini@ior.it
* Correspondence: mmorra@nobilbio.it

1.1 Grape Pomace

Red grape pomace was purchased from a local wine producer (ALEMAT, Penango, AT, Italy). Grape varietal is Croatina (*Vitis Vinifera*), harvested in mid-September 2023. Grape has been harvested by hand and then grapes barriers were removed from the stems and crushed to release the juice. After that the juice, skins and seeds together in stainless steel tanks start the fermentation. After 21 days, skin and seed were taken out and gently pressed.

The pomace obtained after pressing process, was immediately collected, stored under vacuum at -21 °C until the extraction process starting.

Multiresidual analysis showed level of pesticides under the EU safety level report reported in the following database https://ec.europa.eu/food/plant/pesticides/eu-pesticides-database/start/screen/products/details/38, (results could be provided upon request).

1.2 HPLC Analysis

PRPE from Croatina has been characterized by using the High Performance Liquid Chromatography (HPLC, Shimadzu SLC 40 equipped with Diode array Shimadzu SPD-M40) technique. PRPE was filtered through 0.2 µm cellulose acetate filters and analyzed using Kinetex Biphenyl (100 x 3.0 mm, 2.6 µm) from

Phenomenex (Torrance, CA) operated at 40 °C. The mobile phases consisted of 2% (v/v) acetic acid in water (MPA) and 0.5 % acetic acid in water and acetonitrile (50:50 v/v) (MPB) and were used in the gradient method reported in table 1, at a flow rate of 0.4 mL/min and a total run time of 17 min.

Table S1. Details of the HPLC gradient method used for PRPE analysis**.**

**Time [min]**	**MPA [%]**	**MPB [%]**
0 – 3	100 → 70	0 → 30
3 – 8	70 → 40	30 → 60
8 -11	40 → 100	60 → 100
11 - 13	0	100
13 - 14	0 → 70	100 → 30
14-16	70 → 100	30 → 0
16-17	100	0

The injection volume was 3 µL and the diode array operated at the wavelength range 200 to 600 nm. Main polyphenols (Phs) were identified through comparison with reference compounds. The quantitation of individual Phs was performed by using calibration curves of the corresponding reference compounds. Gallic acid, catechin, tannic acid, procyanidin B2, epicatechin, epigallocatechin gallate (280 nm), quercetin (370 nm), myricetin (375 nm), quercitrin (349 nm), rutin (355 nm), kaempferol, isorhamnetin (366 nm), caffeic acid (310 nm), trans p-cumaroyl tartaric acid (313 nm), quercetin 3-glucuronide (354 nm) and malvidin-3-glucoside (520 nm) were dissolved in ethanol : water solution at the concentrations of 5, 25, 50, 100, 150 and 200 µg/mL and analyzed with the same method reported above. The quantitation was performed by applying the standard calibration curve for each standard.

Below, in Figure S1, is represented the chromatograms at different wavelength and in table S1 the quantification of Phs is listed.

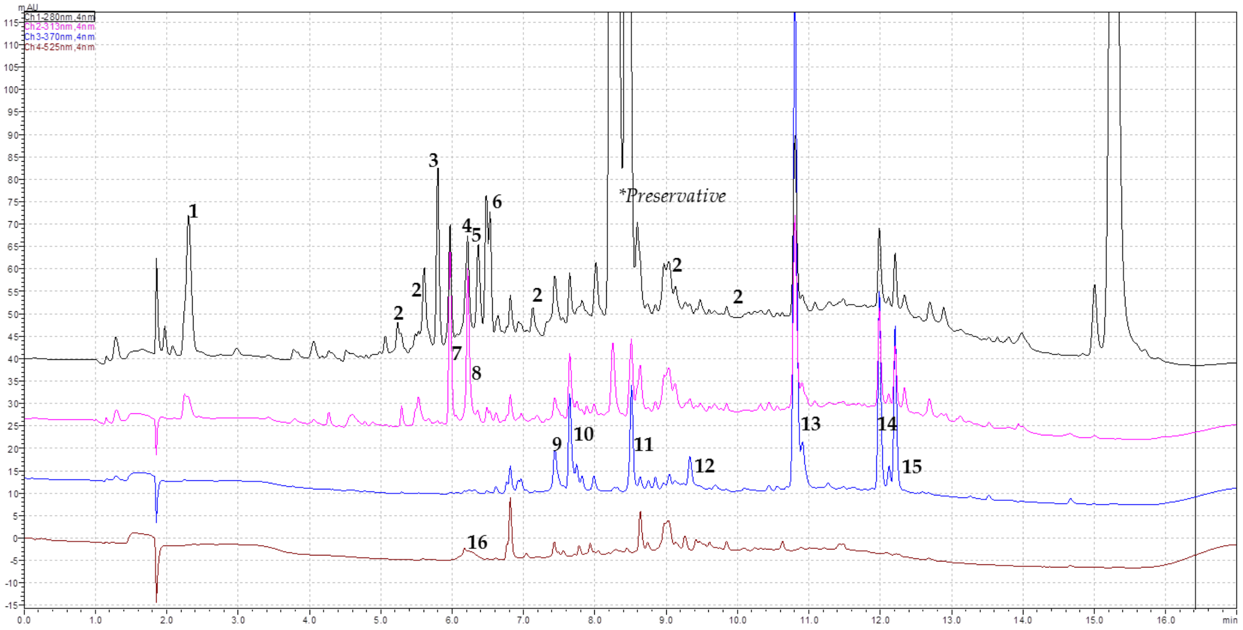

Figure S1. HPLC analysis of PRPE of Croatina. *Chromatograms at different wavelengths (280 nm, 320 nm, 370 nm and 525 nm). The following polyphenols were identified: 1. Gallic acid, 2. Tannic acid, 3. Catechin, 4. Procyanidin B2, 5. Epicatechin, 6. Epigallocatechin gallate , 7. Trans p-coumaroyl tartaric acid, 8. Caffeic acid 9. Rutin, 10. Quercetin-3- glucuronide, 11. Quercitrin, 12. Myricetin, 13. Quercetin, 14. Kampferolo, 15. Isorhamnetin, 16. Malvidin 3-glucoside*

Table S2. Polyphenolic quantification through HPLC-DAD

**Reference number**	**Phs**	**[µg/mL]**
1	Gallic acid	33,03 ± 3.97
2	Tannic acid	1086,04 ± 156.14
3	Catechin	70,90 ± 1.99
4	Procyanidin B2	98,14 ± 5.83
5	Epicatechin	55,69 ± 4.72
6	Epigallocatechin gallate	44.37 ± 1.24

7	Trans p-coumaroyl tartaric acid	23.48 ± 0.69
8	Caffeic acid	15.99 ± 0.25
9	Rutin	40.79 ± 2.89
10	Quercetin-3-glucuronide	46.21 ± 1.32
11	Quercitrin	103,41 ± 5.14
12	Myricetin	13,92 ± 2.87
13	Quercetin	61,76 ± 11.30
14	Kaempferol	38.39 ± 6.67
15	Isorhamnetin	74.11 ± 20.56
16	Malvidin-3-glucoside	2,04 ± 0.75
Total amount of polyphenols identified		1.79 mg/mL

PRPE: polyphenols-rich pomace extract

1.3 Scanning Electron Microscopy Analysis

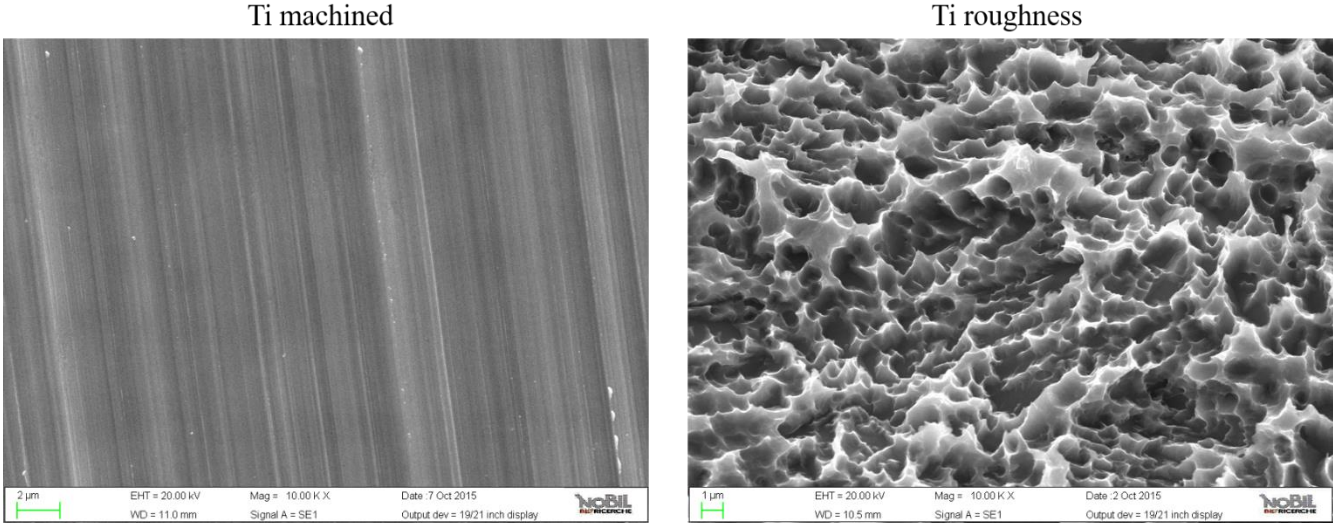

Figure S2. SEM images (10000 x) showing the surface topography of machined (Ti machined) and doubly acid etched (DAE) (Ti roughness) titanium screws used in implant studies

1.4 X-ray photoelectron microscopy

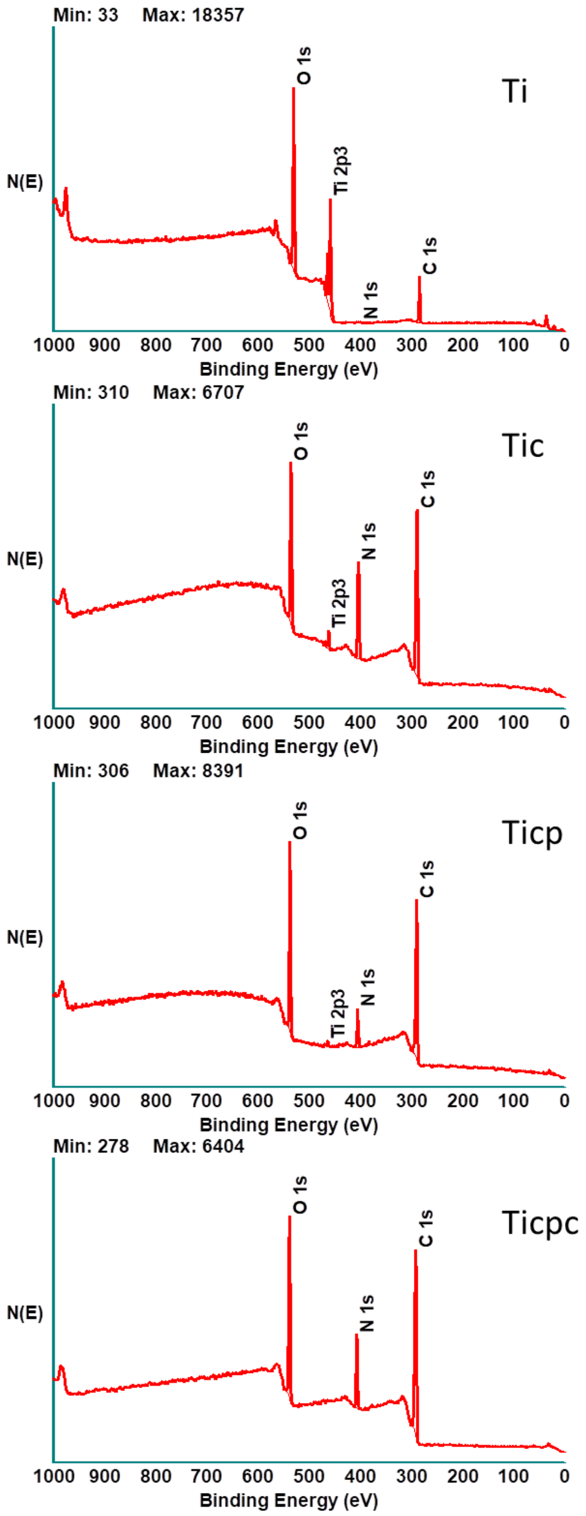

Figure S3. XPS survey spectra of Ti disks after the subsequent steps of samples preparation

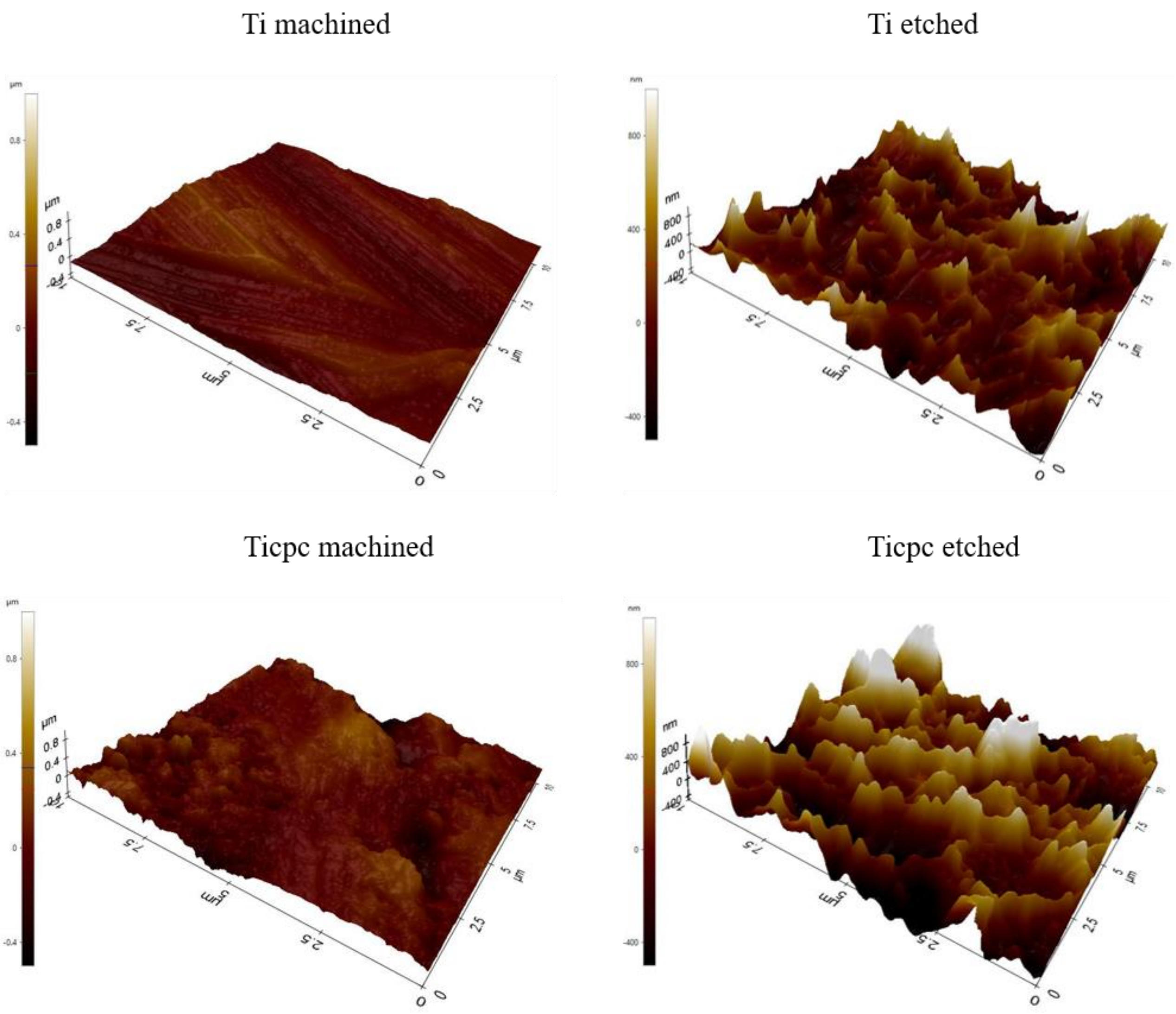

Figure S4. AFM three dimensional images of Ti disk machined and etched, before and after functionalization (Ticpc)
